# Poverty Dynamics and Caries Status in Young Adolescents

**DOI:** 10.1111/cdoe.13012

**Published:** 2024-10-10

**Authors:** Gisselle Carbajal Rodriguez, Agatha W. van Meijeren‐van Lunteren, Eppo B. Wolvius, Lea Kragt

**Affiliations:** ^1^ The Generation R Study Group Erasmus MC University Medical Center Rotterdam The Netherlands; ^2^ Department of Oral and Maxillofacial Surgery, Special Dental Care and Orthodontics Erasmus MC University Medical Center Rotterdam The Netherlands

**Keywords:** adolescents, children, cohort study, dental caries, poverty

## Abstract

**Objectives:**

To investigate whether timing, accumulation and trajectories of poverty are associated with dental caries in young adolescents.

**Methods:**

The study was conducted within the Generation R Study, which is an ongoing population‐based prospective cohort study conducted in Rotterdam, the Netherlands. This study included 2653 children. Information about household income and number of children and adults living in a household at six time points from pregnancy to 13 years old was retrieved from parental questionnaires to construct the poverty variable. Dental caries was assessed with the decayed, missing and filled teeth index through intraoral photographs at the age of 13 years. Sociodemographic and oral health‐related characteristics were included as possible confounders. The association between poverty and dental caries was analysed on the basis of the three lifecourse theories, that is, critical period, cumulative risk and social mobility model. For the latter, we used latent class growth analysis (LCGA) to identify poverty trajectories over time. Next, the associations were studied with Hurdle Negative Binomial Models.

**Results:**

Poverty at birth and intermittent poverty up to the age of 13 were significantly associated with dental caries at 13 years of age (OR 1.41, 95% CI 1.01–1.99; OR 1.36, 95% CI 1.01–1.83 respectively) and with an increased mean number of decayed teeth by 34% (95% CI 1.02–1.76; 95% CI 1.05–1.71, respectively). LCGA showed four trajectories for the probabilities of poverty. All trajectories were significantly associated with dental caries at 13 years of age, with the ‘downward mobility’ trajectory showing the strongest association with dental caries (OR 1.55, 95% CI 1.05–2.29) and an increasing mean number of decayed teeth by 58% (95% CI 1.18–2.12) than the ‘stable absent’ trajectory.

**Conclusion:**

Poverty at birth, intermittent poverty and downward poverty trajectory were associated with higher odds and higher mean number of decayed teeth at 13 years of age. The three lifecourse models influenced dental caries status during adolescence, hence strategies and policies targeted to improve socioeconomic conditions on deprived children should be implemented.

## 1 Introduction

Poverty is a major sociodemographic factor that influences people's health [1]. Likewise, poverty adversely affects oral health [2, 3, 4]. Deprivation during childhood impacts children's nutrition, parental knowledge and attitudes, increasing the risk of a higher prevalence and severity of oral diseases. In the long term, this can lead to pain, infection and a negative effect on oral health‐related quality of life [4]. However, most of the studies conducted only use cross‐sectional measures to assess the influence of income on oral health. Research shows that the development of diseases is based on a continuum of exposures, therefore studying the dynamics of poverty throughout life could provide insights into the complexity of oral diseases and conditions [5].

The lifecourse theory and its influence on health conditions have been extensively explained [5, 6, 7]. In summary, exposure to adverse factors is continual and cumulative throughout life (cumulative risk model). For example, living in low socioeconomic circumstances for a longer time period may impose an increased risk of contracting many diseases [5, 6, 8]. The accumulation may occur gradually, or there may be certain critical or sensitive periods (critical period model) during which people are more vulnerable to developing a disease. Furthermore, changes in social class (social mobility model) give rise to differences in health and disease profiles [6, 7, 8].

The three causal models suggest that the health of individuals depends on the interaction of various protective and risk factors related to behavioural, biological, psychological and environmental influences throughout life [9]. Clinically, tooth decay is caused by deficient oral hygiene and high intake of free sugars [10, 11]. However, it is important to acknowledge that oral health‐related behaviours are socially patterned, and these behaviours are not the only reason explaining different caries levels among the population [11, 12]. Both oral hygiene and nutrition are also subject to habit formation, and so far, it remains unknown whether changes in socioeconomic status (SES) may influence oral health‐related behaviours and subsequently oral health status.

Only a few existing studies use different lifecourse theories on dental caries [13, 14, 15, 16, 17, 18, 19]. Their findings show associations between poverty and unfavourable socioeconomic circumstances during childhood and dental caries later in life [13, 16, 17, 18]. These studies also suggest a dose–response relationship between the number of periods in social disadvantage and dental caries [13, 14, 16, 19]. However, there is no conclusive evidence of whether social mobility during childhood has an impact or not on dental caries in young people with early permanent dentition.

The aim of this study was to investigate whether the timing and accumulation of periods in poverty are associated with dental caries in young adolescents. Furthermore, trajectories of poverty along the 13 years were determined and studied in relation with dental caries status at the age of 13 years.

## 2 Methods

This study is embedded in the Generation R Study, which is an ongoing population‐based prospective cohort study from fetal life onwards, conducted in Rotterdam, the Netherlands. The Medical Ethics Committee of Erasmus Medical Centre approved this research (MEC 2015‐749‐NL55105.078.15). Participants and their parent(s) provided written informed consent before interviews and examinations were performed.

**FIGURE 1:**
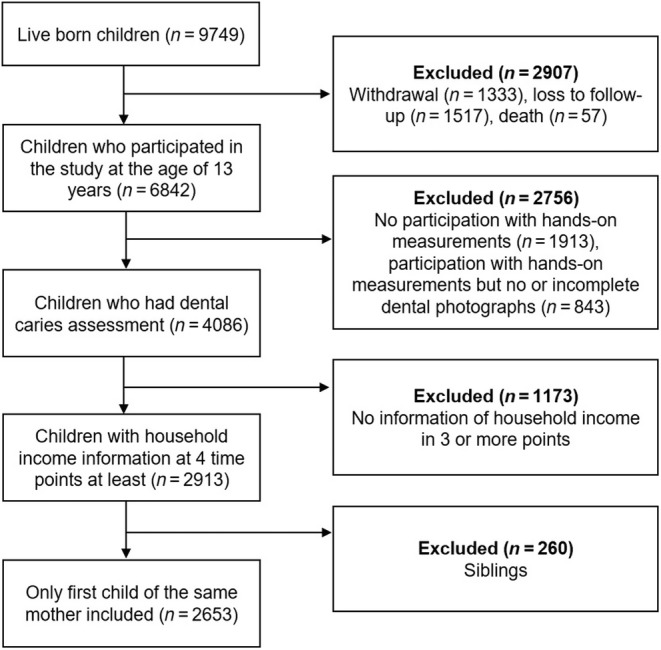
Flowchart of the study population selection.

The Generation R Study is multi‐disciplinary and focusses on diverse health outcomes from early life onward. Pregnant women registered as inhabitants in the municipality of Rotterdam between April 2002 and January 2006, were eligible to participate in the study. In total, 9778 mothers were enrolled at the start of the study and gave birth to 9749 live‐born children [20]. For the current study, data collection took place during pregnancy (early, mid and late), childhood and early adolescence. During pregnancy and when the children were 2, 3, 6, 9 and 13 years old, information regarding household income was retrieved. At the age of 13 years, 6842 children participated in the study, and dental caries in the permanent dentition was assessed in 4086 children. Children who provided information about net household income at four time points at least (out of six) were included in the analysis (*n* = 2913). In addition, siblings were excluded; therefore, the final study population comprised 2653 children (Figure 1).

### 2.1 Dental Caries

Intraoral photographs were taken to all participants who visited the research centre at the follow‐up phase of children aged 13 years. Children were instructed to brush their teeth for 2 min. A quantitative light fluorescence camera (Qraycam Pro; Inspektor Research Systems BV) was used to capture children's dentition in at least five white light and blue light photographs. The intraoral photographs were scored for dental caries by two trained researchers. Ten per cent of the participants were selected at random and evaluated double to calculate the intra‐rater reliability (weighted kappa = 0.94) and inter‐observer reliability (weighted kappa = 0.84) and both exhibited high agreement. The reliability of the quantitative light fluorescence camera for the assessment of the decayed, missing and filled teeth (DMFT) index was evaluated, and it showed good sensitivity and high specificity compared with the clinical visual tactile inspection [21]. Dental caries in the permanent dentition was assessed at the age of 13 using the DMFT index [22]. Decayed teeth were scored from caries with visible enamel breakdown, which could be observed by white spot lesions and brown carious discoloration. Missing teeth were scored when elements were missing solely because of caries, which was verified on dental panoramic radiographs taken at the age of nine. Fillings were scored when teeth were restored because of caries.

### 2.2 Poverty

Questionnaires were used to collect data regarding household income. A multiple‐choice question asked parents to indicate the net household income category at six time points (during pregnancy and at the child ages of 2, 3, 6, 9 and 13 years). Net household income included monthly income from work, benefits, and/or income from assets that respondents received in‐hand following the deduction of tax and other contributions. Parents were also asked about the number of adults and children in the household (i.e., the number of units) living from this income. The mean income to each income category was calculated (e.g., 3200 euros for the category receiving 2800–3600 euros per month), and then, those figures were used to calculate the equivalised disposable income, based on the modified scale from the Organisation for Economic Co‐operation and Development (OECD). Children living in a household with an income below the European poverty threshold—which is less than 60% of the national median (equivalised) disposable income—were assigned as ‘poor’ [23]. As data collection at each phase took place over several years, the median year of the years included in each phase was used. Two missing measurements of poverty were considered as not poor. When data on income were missing more than four time points, children were excluded from the analyses.

The following variables were defined on the basis of the poverty status. Poverty at birth and poverty at 2 years old were defined as yes/no variables. Cumulative poverty was defined by the number of episodes of poverty between pregnancy and the child's age of 13 years: no poverty (zero episodes of poverty), intermittent poverty (one–three episodes of poverty), or chronic poverty (four–six episodes of poverty). The poverty trajectories over time were identified using LCGA. This method assigns participants to the trajectory group to which they had the highest probability of belonging based on similar patterns of observed repeated measurement [24]. The lowest Bayesian information criterion (BIC) value was used to select the number of trajectories. LCGA was carried out with two to six classes. Then, a categorical variable including all the trajectories was created as a predictor for assessing dental caries.

### 2.3 Covariates

A number of characteristics were considered as confounders in the analyses such as age, gender and maternal age at enrolment. In addition, maternal educational level was retrieved using questionnaires at the age of 6 years and recategorised as low, middle and high. Children's ethnic background was defined according to the Dutch classification of ethnic background as ‘Dutch’ and ‘non‐Dutch’ if one of the parents was born in another country than the Netherlands [25]. Financial stress was retrieved from questionnaires at the age of 13 years, and it indicated whether the family had experienced worries or tensions in the past 2 years because of financial difficulties. Oral health factors were assessed using questionnaires at the age of 13 years. Sugar intake included two questions about sweet and chocolate frequency consumption and about soft drinks on a weekly basis. For the analyses, sugar intake was categorised as ‘low’ (≤ 2 sugar‐containing items a day) and ‘high’ (≥ 3 sugar‐containing items a day). Toothbrushing frequency was considered as ‘< 2 per day’ and ‘≥ 2 per day’. Dental visits in the last 12 months were assessed with Yes/No.

### 2.4 Statistical Analysis

Descriptive statistics of the study population were presented. Because the DMFT value is zero‐inflated and over‐dispersed, negative binomial hurdle regression (NBHR) models were used to study the association of poverty and dental caries at the age of 13 years. A hurdle model output consists of two parts: a zero‐hurdle part equal to binomial logistic regression that estimates the OR of having caries experience, and a count hurdle part which estimates the contribution of poverty to the amount of caries experience using the rate ratio (RR) of the mean caries counts [26]. Three models were built: the first model included child's gender and age; the second model additionally adjusted for sociodemographic indicators (maternal education level, maternal age at enrolment, children's ethnic background and financial stress); and the third model additionally accounted for oral health factors. Collinearity among determinants was tested and was absent. LCGA was performed in Mplus version 8.6. The statistical analyses were carried out using R version 4.3.2 for Windows (R core team, Vienna, Austria). Multiple imputation was performed in 10 data sets to account for information bias related to missing data using the ‘mice’ package, but the exposure and outcome were not imputed [27]. For all the analyses, the significance level was set at 0.05.

## 3 Results

This study analysed 2653 children most of whom had Dutch ethnic background (78.2%) with highly educated mothers. The distribution of poverty showed that 9.4% of the study population was born into poverty. Up to the age of 13 years, 3.9% of the children had experienced four or more episodes of poverty. Regarding oral health factors, 33.4% of the adolescents had dental caries, and most of the participants had an adequate oral health care (Table 1).

**TABLE 1:** Characteristics of the sample by dental caries status (*N* = 2653).

	Missing data	DMFT = 0	DMFT ≥ 1	
	(*n*, %)	(*n* = 1767)	(*n* = 886)	*p*‐value
**Sociodemographics**				
Gender				0.009
Male		881 (49.9)	394 (44.5)	
Female		886 (50.1)	492 (55.5)	
Age (SD)		13.57 (0.29)	13.62 (0.36)	< 0.001
Maternal age at enrolment (SD)		31.92 (4.3)	31.53 (4.8)	0.02
Maternal education level	87 (3.3)			< 0.001
Low		137 (8.0)	157 (18.4)	
Middle		479 (27.9)	237 (27.8)	
High		1098 (64.1)	458 (53.8)	
Children's ethnic background	2 (0.1)			< 0.001
Dutch		1442 (81.6)	631 (71.4)	
Non‐Dutch		325 (18.4)	253 (28.6)	
Financial stress at home (yes)	234 (8.8)	185 (11.4)	113 (14.2)	0.047
Poverty during pregnancy (yes)	401 (15.1)	98 (6.5)	113 (15.1)	< 0.001
Poverty at 2 years	376 (14.2)	88 (5.7)	87 (11.7)	< 0.001
Poverty at 3 years	464 (17.5)	78 (5.4)	87 (12.4)	< 0.001
Poverty at 6 years	210 (7.9)	88 (5.4)	94 (11.5)	< 0.001
Poverty at 9 years	149 (5.6)	121 (7.2)	117 (14.0)	< 0.001
Poverty at 13 years	315 (11.9)	109 (6.9)	99 (12.9)	< 0.001
Cumulative poverty				< 0.001
0 episodes (no poverty)		1580 (89.4)	727 (82.1)	
1,2,3 episodes (intermittent)		125 (7.1)	118 (13.3)	
4,5,6 episodes (chronic poverty)		62 (3.5)	41 (4.6)	
**Oral health characteristics**				
Toothbrushing frequency	182 (6.9)			< 0.001
< 2 times per day		253 (15.2)	186 (23.0)	
≥ 2 times per day		1409 (84.8)	623 (77.0)	
Dental visit last year (yes)	187 (7.0)	1623 (97.9)	795 (98.4)	0.40
Sugar intake	199 (7.5)			0.26
≤ 2 items per day		1571 (95.0)	751 (93.9)	
≥ 3 items per day		83 (5.0)	49 (6.1)	

*Note:* Table based on a non‐imputed dataset. Missing values of confounders were imputed when performing the NBHR analyses. Two missing measurements of poverty were considered as ‘not poor’. *p*‐values estimated using χ2 tests for categorical variables and independent *t*‐test for continuous variables.

Table 2 shows that after adjustment of potential confounders, poverty at birth was significantly associated with dental caries at the age of 13 years (OR 1.41, 95% CI 1.01–1.99). Poverty at birth also significantly increased the mean number of teeth affected by dental caries (RR 1.34, 95% CI 1.02–1.76) compared with participants who were not born into poverty. On the contrary, poverty at 2 years old was not significantly associated with dental caries. Intermittent poverty was associated with any dental caries after correction for sociodemographic factors (OR 1.36, 95% CI 1.01–1.83) and with the mean number of decayed teeth (RR 1.34, 95% CI 1.05–1.71). In contrast, chronic poverty was not associated with dental caries at 13 compared with children who were never poor.

**TABLE 2:** Association between both poverty at critical periods and cumulative poverty with dental caries.

		Model A		Model B		Model C	
	*N*	Zero part OR (95% CI)	Hurdle part RR (95% CI)	Zero part OR (95% CI)	Hurdle part RR (95% CI)	Zero part OR (95% CI)	Hurdle part RR (95% CI)
**Poverty at birth**							
No	2041	Ref	Ref	Ref	Ref	Ref	Ref
Yes	211	**2.37 (1.81–3.11)**	**1.73 (1.37–2.13)**	**1.50 (1.09–2.07)**	**1.40 (1.04–1.83)**	**1.41 (1.01–1.99)**	**1.34 (1.02–1.76)**
**Poverty at 2 years old**							
No	2102	Ref.	Ref.	Ref.	Ref.	Ref.	Ref.
Yes	175	**2.06 (1.51–2.81)**	**1.51 (1.16–1.97)**	1.24 (0.88–1.75)	1.14 (0.87–1.50)	1.12 (0.77–1.61)	1.09 (0.88–1.44)
**Cumulative poverty**							
No poverty	2307	Ref	Ref	Ref	Ref	Ref	Ref
Intermittent poverty	243	**2.01 (1.54–2.63)**	**1.63 (1.29–2.05)**	**1.36 (1.01–1.83)**	**1.36 (1.07–1.74)**	1.31 (0.97–1.77)	**1.34 (1.05–1.71)**
Chronic poverty	103	1.44 (0.96–2.16)	**1.47 (1.01–2.13)**	0.98 (0.65–1.54)	1.23 (0.85–1.78)	0.99 (0.64–1.53)	1.20 (0.83–1.74)

*Note:* Model A: adjusted for gender and age. Model B: model A + adjusted for maternal age, maternal educational level, ethnicity, financial stress. Model C: model B + adjusted for toothbrushing, dental visits, sugar consumption. Poverty at birth and poverty at 2 years old were additionally adjusted for the other time point. Bold values represent statistical significant difference.

**FIGURE 2:**
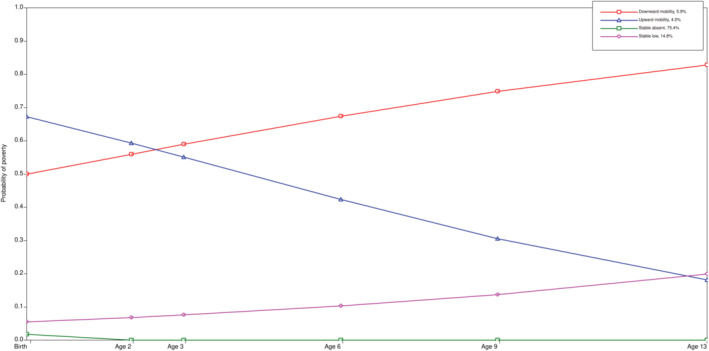
Probability of poverty in four trajectories from birth until the age of 13 years (*n* = 2653): ‘stable absent’ 75.4%, ‘stable low’ 14.8%, ‘upward mobility’ 4%, ‘downward mobility’ 5.9%.

A four‐trajectory model showed the best fit for the data (Table S1). Based on the probability of poverty, the following four trajectories were identified: ‘stable absent’ 75.4% (i.e., participants who remain out of poverty), ‘stable low’ 14.8% (i.e., those who continue in a low probability), ‘upward mobility’ 4% (i.e., participants with a high probability of poverty but after a steady decline ended up with a low probability) and ‘downward mobility’ 5.9% (i.e., those whose likelihood of poverty increased steadily over time). No trajectories characterising a constant probability of poverty (always poor) were identified (Figure 2).

Table 3 shows a significant association between poverty trajectories and dental caries at the age of 13 after fully adjustment. Compared with the ‘stable absent’ trajectory, all the other trajectories had either higher odds of dental caries (‘upward mobility’ OR 1.62, 95% CI 1.03–2.56) or higher mean number of decayed teeth (‘stable low’ RR 1.66, 95% CI 1.24–2.24). Furthermore, ‘downward mobility’ trajectory was significantly associated with both, dental caries and mean number of decayed teeth (OR 1.55, 95% CI 1.05–2.29; RR 1.58 95% CI 1.18–2.12).

**TABLE 3:** Associations between poverty trajectories and dental caries.

		Model A		Model B		Model C	
	*N*	Zero part OR (95% CI)	Hurdle part RR (95% CI)	Zero part OR (95% CI)	Hurdle part RR (95% CI)	Zero part OR (95% CI)	Hurdle part RR (95% CI)
**Poverty trajectory**							
Stable absent	2216	Ref	Ref	Ref	Ref	Ref	Ref
Stable low	194	1.15 (0.84–1.57)	**1.81 (1.36–2.40)**	0.87 (0.62–1.22)	**1.69 (1.26–2.28)**	0.87 (0.61–1.22)	**1.66 (1.24–2.24)**
Upward mobility	98	**2.65 (1.76–4.00)**	1.30 (0.94–1.80)	**1.66 (1.06–2.60)**	1.12 (0.79–1.59)	**1.62 (1.03–2.56)**	1.10 (0.77–1.56)
Downward mobility	145	**2.59 (1.85–3.64)**	**1.98 (1.53–2.60)**	**1.58 (1.08–2.33)**	**1.65 (1.23–2.20)**	**1.55 (1.05–2.29)**	**1.58 (1.18–2.12)**

*Note:* Model A: adjusted for gender and age. Model B: model A + adjusted for maternal age, maternal educational level, ethnicity, financial stress. Model C: model B + adjusted for toothbrushing, dental visits, sugar consumption. Bold values represent statistical significant difference.

## 4 Discussion

Consistent with lifecourse theories, this study found that dental caries in young adolescents is associated with poverty during the time of birth and with intermittent poverty from birth up to the age of 13 years. Furthermore, downward mobility in poverty was associated with higher odds of dental caries and an increased mean number of decayed teeth.

This prospective cohort study analyses poverty status through several repeated measurements over 13 years and dental caries in young adolescents. Extensive existing literatures underline the importance of the first 1000 days of life [28, 29]. Therefore, it was expected that poverty at birth could be a risk factor associated with caries development later in life. A birth cohort study assessed the relationship between poverty at birth and caries status, with findings consistent with the present results. Peres et al. [16] reported a significant positive association between poverty at birth and the number of unsound teeth in adults. Other longitudinal studies evaluated the importance of other ‘critical periods’ in the prevalence dental caries and diverse oral health outcomes, but findings were mixed [13, 14, 16, 17, 18, 19, 30]. For instance, four studies found that SES measured during adulthood may have a stronger relationship with adult oral health status than SES measured during childhood or adolescence [13, 14, 16, 30]. Findings from another longitudinal study suggest that the strong association between SES in early life and later oral health outcomes, seems to be indirect effects. An early created socioeconomic gap acts as a chain of risks throughout the lifecourse [31]. Thus, although this study could not analyse the effect from early life on dental caries in adulthood, the results indicate the importance of early influences on later oral health that should not be neglected.

Although the best predictor for dental caries is past caries experience [32], research suggests that children who experienced early disadvantage, but later see an improvement in their SES, may have lower risk of caries in their permanent teeth [33]. Likewise, other studies using different oral health outcomes found the same positive effect of upward social mobility, which suggests that proximal time points in the trajectories may have a more important effect on young or adult oral health [34]. Findings from this research are in agreement with those, indicating that an upward trajectory did not completely attenuate the negative effects of deprivation during early childhood or adolescence on adult dental health [15, 17, 35]. In this study, participants with a favourable change in their household income were more likely to have dental caries than those who remain in the stable absent trajectory. Unhealthy oral health behaviours such as a lack of toothbrushing, dental check‐ups and a high intake of free sugars are developed and established during early years. These may be carried through into adolescence, despite an improvement in a family's SES [12, 13]. This study found that downward mobility in poverty was associated with dental caries, and it increased the mean number of decayed teeth at 13 years of age, which is in line with previous studies [14, 18]. Downward mobility may reduce the likelihood of a child attending a dental service and therefore may not receive treatment. Despite children's dental treatment in the Netherlands is reimbursed by the health insurance, deprived families may not be aware of children's oral health needs or they may prioritise other family issues. In addition, it has been found in the literature that psychological factors could influence oral health [36, 37].

In contrast with previous research [13, 19], the findings of the present study showed that there was not a graded relationship between the number of episodes of poverty and dental caries. Poverty measurement in six different time points over 13 years was included, whereas most of the studies included only two or three time points. However, intermittent poverty was associated with decayed teeth. This can be explained by the fact that along the six time points assessed, some time periods in the lifecourse are potentially more crucial than others [14]. Furthermore, the analysis showed that the chronically poor were not significantly affected by dental caries. This may be down to a lack of power, as the number of children in chronic poverty was low. In addition, existing literature showed that, in younger generations, with good oral health, there was no evidence of a gradient when inequalities were estimated [38]. Along with this, it may be possible that the most vulnerable may have received additional support from institutions which could diminish the socioeconomic difference found in this study.

Regarding the strengths of this study, household income was used as a measure of poverty which is an accurate indicator [39]. Poverty was investigated in six time points in 13‐year‐old‐adolescents; therefore, it likely represents the whole set of socioeconomic conditions that adolescents experienced across their lifespan. Due to the extensive data collection within this cohort, the authors were able to adjust the analysis for several potential confounders considered in the previous literature. However, residual confounding remains an issue. In terms of limitations in this study, it is acknowledged that dental caries was assessed using intraoral photographs which may be more difficult to diagnose between certain stages of caries development when compared to clinical assessment, which may result in an underestimation of the condition. Finally, these findings must be considered with caution and cannot be generalised to all populations [9]. Differences in terms of public health policies related to access to dental services, legislation about products high in free sugars and water fluoridation availability may influence the strength of the association between tooth decay and poverty. For future research, it is recommended to examine different critical periods in the lifecourse of the study population and relate these with the socioeconomic trajectories. Efforts should be made to analyse and report findings following the recommendations of the Oral Health‐Related Birth Cohort Studies Consortium [40].

## 5 Conclusions

This study found an association between experiencing poverty at a critical period early in life such as birth, intermittent poverty from birth until the age of 13 years and dental caries at 13 years. Moreover, downward mobility was also associated with decayed teeth at the age of 13 years. Lifecourse models influence dental caries across childhood and adolescence, and it is important to monitor vulnerable population and develop strategies targeted on deprived children from their early years onward.

## Conflicts of Interest

The authors declare no conflicts of interest.

## Supporting information


Table S1.

## Data Availability

The data that support the findings of this study are available on request from the corresponding author. The data are not publicly available due to privacy or ethical restrictions.
