# Supplementary material for: Poverty Dynamics and Caries Status in Young Adolescents
Source: Community Dent Oral Epidemiol. 2024 Oct 10;53(1):90–7. doi: 10.1111/cdoe.13012 (PMC11754150; doi:10.1111/cdoe.13012)
Supplement: Supplementary file 1 — Table S1. [file CDOE-53-90-s001.docx]

**Supplement Table 1 . Model-fit indices for alternative latent class analyses for poverty trajectories**

|  | **Number of classes** | | | | |
| --- | --- | --- | --- | --- | --- |
| Model-fit indices | **Two** | **Three** | **Four** | **Five** | **Six** |
| Number of parameters | 5 | 8 | 11 | 14 | 17 |
| Log likelihood | -4200 | -3122 | -3088 | -3096 | -3088 |
| **BIC** | 6283 | 6265 | 6263 | 6278 | 6308 |
| LMR test | **<0.001** | **<0.001** | **<0.001** | **<0.001** | 0.3217 |

BIC: Bayesian Information Criterion

LMR: Lo-Mendel-Rubin statistics
